# Tumor-derived exosomes promote tumor progression and T-cell dysfunction through the regulation of enriched exosomal microRNAs in human nasopharyngeal carcinoma

**DOI:** 10.18632/oncotarget.2118

**Published:** 2014-06-19

**Authors:** Shu-biao Ye, Ze-Lei Li, Dong-hua Luo, Bi-jun Huang, Yu-Suan Chen, Xiao-shi Zhang, Jun Cui, Yi-xin Zeng, Jiang Li

**Affiliations:** ^1^ State Key Laboratory of Oncology in South China, Sun Yat-Sen University Cancer Center, Guangzhou, China; ^2^ Collaborative Innovation Center of Cancer Medicine, Sun Yat-Sen University Cancer Center, Guangzhou, China; ^3^ Department of Biotherapy, Sun Yat-Sen University Cancer Center, Guangzhou, China; ^4^ Department of Nasopharyngeal Carcinoma, Sun Yat-Sen University Cancer Center, Guangzhou, China; ^5^ Department of Radiotherapy, Sun Yat-Sen University Cancer Center, Guangzhou, China; ^6^ Key Laboratory of Gene Engineering of the Ministry of Education, State Key Laboratory of Biocontrol, College of Life Sciences, Sun Yat-sen University, Guangzhou, China

**Keywords:** exosomes, nasopharyngeal carcinoma, tumor microenvironment

## Abstract

Tumor-derived exosomes contain biologically active proteins and messenger and microRNAs (miRNAs). These particles serve as vehicles of intercellular communication and are emerging mediators of tumorigenesis and immune escape. Here, we isolated 30-100 nm exosomes from the serum of patients with nasopharyngeal carcinoma (NPC) or the supernatant of TW03 cells. Increased circulating exosome concentrations were correlated with advanced lymphoid node stage and poor prognosis in NPC patients (P < 0.05). TW03-derived exosomes impaired T-cell function by inhibiting T-cell proliferation and Th1 and Th17 differentiation and promoting Treg induction by NPC cells *in vitro*. These results are associated with decreases in ERK, STAT1, and STAT3 phosphorylation and increases in STAT5 phosphorylation in exosome-stimulated T-cells. TW03-derived exosomes increased the proinflammatory cytokines IL-1β, IL-6, and IL-10 but decreased IFNγ, IL-2, and IL-17 release from CD4^+^ or CD8+ T-cells. Furthermore, five commonly over-expressed miRNAs were identified in the exosomes from patient sera or NPC cells: hsa-miR-24-3p, hsa-miR-891a, hsa-miR-106a-5p, hsa-miR-20a-5p, and hsa-miR-1908. These over-expressed miRNA clusters down-regulated the MARK1 signaling pathway to alter cell proliferation and differentiation. Overall, these observations reveal the clinical relevance and prognostic value of tumor-derived exosomes and identify a unique intercellular mechanism mediated by tumor-derived exosomes to modulate T-cell function in NPC.

## INTRODUCTION

Nasopharyngeal carcinoma (NPC) is an Epstein-Barr virus (EBV)-associated malignancy with a complex etiology involving viral, environmental, and hereditary factors [1-4]. EBV latent type II antigens, including Epstein-Barr nuclear antigen 1 (EBNA1), latent membrane proteins 1 and 2 (LMP1 and LMP2), and BARF1, are consistently expressed in NPC cells [5]. The appearance of a malignant process producing several immunogenic viral proteins within a context of local inflammation and heavy leukocytic infiltration is one major paradox of NPC pathogenesis. Moreover, another important biologic feature of NPC is the presence of a massive population of tumor-infiltrating lymphocytes (TILs) in the primary tumor [6-8]. Our previous study indicated that the frequencies of different TIL subsets, including CD8^+^, FOXP3^+^, and IL-17-producing TILs, have prognostic value in NPC patients. Moreover, expansions of regulatory T cells (Tregs) and Th17 cells in NPC tissues were identified in our previous work and that of others [9, 10]. However, the concepts of tumor immune surveillance and tumor immune evasion, which have been debated for more than a century, remain poorly defined.

Exosomes are a population of nanometer-sized vesicles (30-100 nm) actively secreted by a diverse range of living cells and have physiological functions that include immune modulation. Exosomes have a topology identical to that of a cell and contain a broad array of biologically active material including proteins, nucleotides, deoxynucleotides, and non-coding microRNAs (miRNAs) [11-17]. Emerging evidence indicates that exosomes play a key role in tumor-host crosstalk and that exosome secretion, composition, and functional capacity are altered as tumors progress to an aggressive phenotype. In addition to transmitting signals to other cancer cells, the exosomes released by cancer cells can also signal to stromal cells within the cancer microenvironment, thus impacting tumor cell growth, metastasis, and angiogenesis and generating the cancer microenvironment [13, 18-25]. Furthermore, cancer exosomes generally modulate immune responses in a dual manner. First, tumor exosomes closely reflect the parental cancer cells and typically carry tumor antigens specific for the tumors that produce and release them, such as Melan A, HER2, Silv, CEA, mesothelin, CD24, and EpCAM, thereby enhancing tumor antigen recognition and priming cytotoxic T cells to induce protective antitumor immune responses [12, 26-28]. Second, tumor exosomes carry not only tumor markers but also proteins with detrimental effects on the immune system, such as FasL, TRAIL, and PD-L1, which promote apoptosis. Exosomes can selectively impair lymphocyte IL-2 responses while supporting Tregs by inducing and up-regulating their suppressive function through TGF-β and IL-10-dependent pathways. Moreover, there is convincing evidence that the effect of tumor exosomes originating from mammary, lung, colon, prostate, and ovarian cancers is a powerful immune suppression, promoting the establishment and metastatic spread of the primary tumor [29-34].

Here, we investigate the role of tumor-derived exosomes in disease progression and immune regulation in NPC. We observed that the serum exosome concentration was positively correlated with tumor lymphoid node transfer and shorter disease-free survival in NPC patients. Furthermore, we hypothesized that NPC-derived exosomes would suppress T-cell immune responses, and we tested whether these exosomes could modulate T-cell proliferation, differentiation, and cytokine secretion. In addition, we screened for the presence of miRNAs in NPC-derived exosomes and analyzed the biological function of over-expressed exosomal miRNA. Altogether, our results demonstrate that NPC TW03-derived exosomes impair T-cell function *in vitro* by disturbing molecular signaling, including the phosphorylation of ERK and STATs in T-cells, and are associated with an enrichment of exosomal miRNA. Moreover, the level of plasma exosomal protein showed clinical relevance and prognostic value in NPC patients.

## RESULTS

### Increased circulating exosome concentrations were correlated with tumor lymph node metastasis and poor disease-free survival in NPC patients

**Figure 1:**
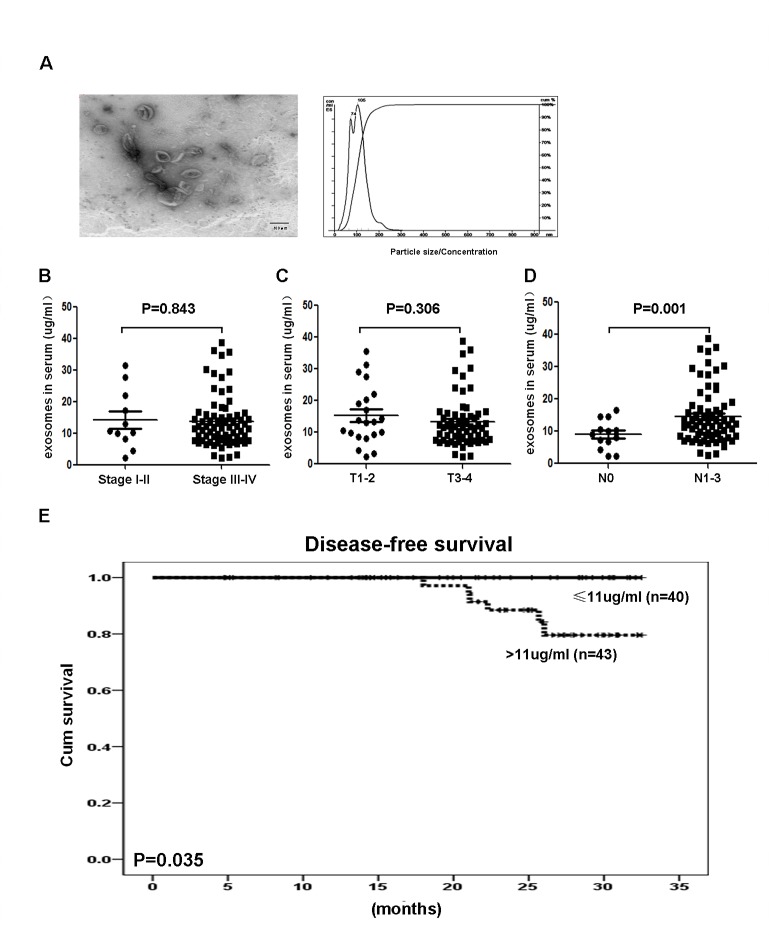
Identification and clinical significance of NPC-derived exosomes A. A representative electron microscopic image of exosomes derived from NPC cells; scale bar, 100 nm. B. Statistical analysis of the correlations between circulating exosomal protein concentrations and clinical parameters, including clinical stage, tumor stage, and lymphoid node stage, and the significant association between the circulating exosomal protein concentration and tumor lymph node metastasis (P = 0.001). C. Kaplan-Meier survival curves showing that the disease-free prognosis of patients with NPC was negatively associated with the circulating exosomal protein concentration (P = 0.035). Stage = clinical stage; T = tumor stage; N = lymphoid node status.

To determine the clinical relevance of circulating exosome concentrations in NPC patients, we prospectively isolated and charac­terized exosomes from the plasma of human subjects with different clinical stages of NPC and the supernatant of NPC TW03 cells. In this study, we defined the exosomes released from NPC TW03 (EBV^+^) or TW03 (EBV^−^) cells as EXO1 or EXO2 and calculated the exosome concentrations using the exosomal protein concentrations. By electron microscopic analysis, the exosomes purified from the serum of NPC patients showed rounded membrane-bound vesicles with an exosome size range of 30-100 nm (Fig. 1A). The presence of known exosome markers, including CD63, LAMP1, major histocompatibility complex class I (MHC-I) and class II (MHC-DR), and HSP70, and other immune-related markers, including EBV latent antigen LMP1 (EXO1), galectin-9 (a ligand of the membrane receptor Tim-3), chemokine receptor CXCR4, and membrane-bound TGF-β, were observed on isolated tumor-derived exosomes. In contrast, the absence of 5'-nucleotidase (CD73), ectonucleoside triphosphate diphosphohydrolase 1 (CD39) and Cytochrome C was observed on these isolated tumor-derived exosomes (Fig. S1).

Furthermore, high levels of exosomal protein (> 11 μg/mL) were positively correlated with tumor lymph node metastasis and a shorter disease-free survival in NPC patients (n = 83, P = 0.001 and 0.035, respectively), as shown in Fig. 1B and C. These data support the notion that circulating exosome concentrations have clinical significance and prognostic value in NPC patients.

### NPC-derived exosomes impeded the proliferation of T lymphocytes and the differentiation of Th1 and Th17 cells but induced the differentiation of Tregs

**Figure 2:**
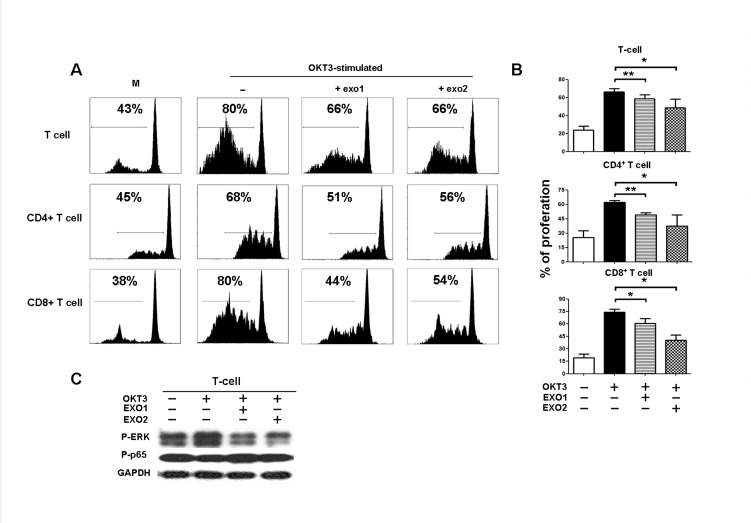
Inhibition of PBMC proliferation by NPC-derived exosomes PBMCs were stained with CFSE, cultured for 5 days, and then stained with monoclonal antibodies against CD4 and CD8; proliferation was quantified as the percentages of CFSE^low^ cells among unfractionated PBMCs, CD4^+^, and CD8^+^ T-cells. A. Representative histogram of the FACS analysis, one of 5 experiments. B. Statistical analysis of T cell proliferation in the presence or absence of exosomes. Columns, mean (n = 5); bars, SE. *, P < 0.05; **, P < 0.01. C. Western blot analysis of P-ERK and P-P65 expression in PBMCs stimulated or unstimulated with OKT3 in the presence or absence of EXOs. P-ERK expression was decreased in the EXO-treated PBMCs compared with PBMCs without EXO treatment; the GAPDH gene was included as a control.

To address whether a high level of circulating exosomal protein in patients with tumor lymph node metastasis is associated with T-cell dysfunction, we examined the effect of NPC TW03-released exosomes on the T-cell-based immune response by analyzing the proliferation and differentiation of T-cells when treated with NPC TW03-derived exosomes *in vitro*. The proliferation of OKT3-stimulated T-cells was significantly decreased when treated with EXO1 or EXO2, including both CD4^+^ T-cells and CD8^+^ T-cells (P < 0.05), as shown in Figure 2A-C. Moreover, treatment with EXO1 or EXO2 decreased the level of ERK phosphorylation but not p65 phosphorylation in OKT3-stimulated T-cells (Fig. 2D).

**Figure 3:**
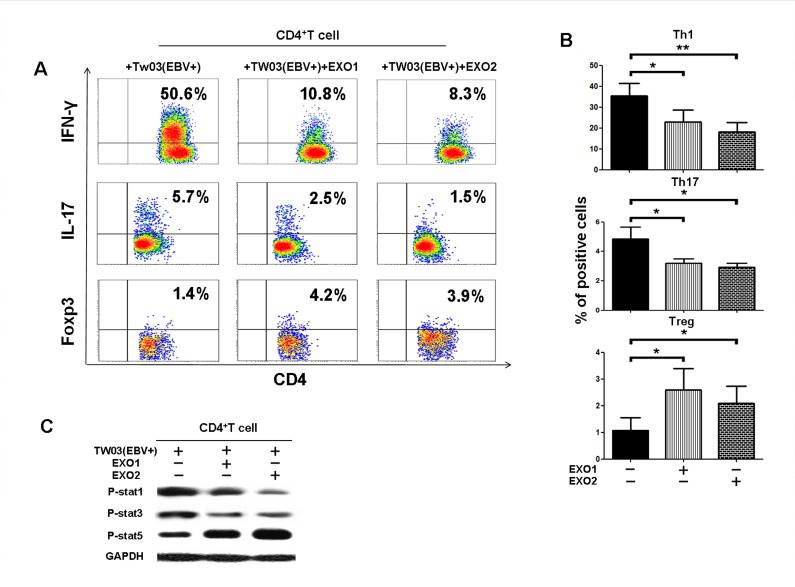
NPC tumor-released exosomes inhibited Th1 and Th17 cell induction but promoted Tregs by altering STAT protein phosphorylation A. Differentiation of naïve CD4^+^ T cells into Th1 and Th17 cells and Tregs after stimulation with tumor cells with or without EXOs. Purified naïve CD4^+^ T cells from healthy donors were co-cultured with the irradiated NPC cell line TW03 (EBV^+^) and then treated with EXOs or left or untreated in the presence of OKT3 in IL-2 medium for 5 days. INFγ, IL-17, and Foxp3 staining following by FACS analysis was performed after stimulation with PMA/ionomycin. Representative data of five experiments are shown. B. Numerous data showing the mean percentage ± S.E.M. of positive cell subsets in three independent experiments. * means P < 0.05. C. Western blot analysis for the expression of P-STAT1, P-STAT3, and P-STAT5 proteins. Stimulation with EXOs decreased the expression of P-STAT1 and P-STAT3 but increased the expression of P-STAT5 in OKT3 and NPC cells stimulated by CD4^+^ T cells; the GAPDH gene was included as a control. Representative data of three experiments are shown.

Our previous study reported that NPC cells can induce the differentiation of Th1 and Th17 cells and Tregs in a coculture system *in vitro* [35]. To evaluate whether NPC exosomes play a role in the T-cell differentiation mediated by NPC cells, we assayed the frequency of IFNγ- and IL-17-producing T cells and FOXP3^+^ Tregs in the CD4^+^ T-cell population by FACS after coculture with TW03 (EBV^+^) cells and EXO1 or EXO2 in IL-2 medium for 7 days *in vitro*. As shown in Figure 3A, the proportion of Th1 (IFNγ-producing cells) and Th17 (IL-17-producing cells) cells was significantly decreased and the proportion of FOXP3^+^ Tregs was significantly increased when CD4^+^ PBMCs were cocultured with TW03 (EBV^+^) cells and EXO1 or EXO2 compared with CD4^+^ PBMCs cocultured with TW03 (EBV^+^) cells alone (Fig. 3A and B). Moreover, the phosphorylation of STAT1 and STAT3 was decreased but the abundance of p-STAT5 was increased in CD4^+^ PBMCs cocultured with TW03 (EBV^+^) cells and EXO1 or EXO2 compared with PBMCs cocultured with TW03 (EBV^+^) cells alone (Fig. 3C). These observations indicate that TW03-derived exosomes exert an effect on the proliferation and differentiation of T-cells by altering the phosphorylation of ERK and STAT proteins in T cells.

### Tumor-derived exosomes altered the cytokine profiles of stimulated lymphocytes from NPC specimens

**Figure 4:**
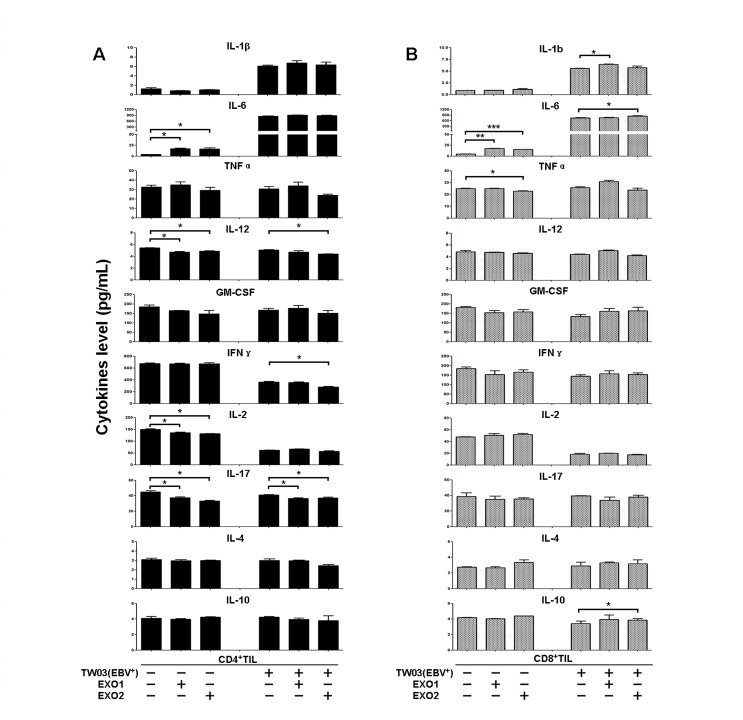
Cytokine secretion by CD4^+^ and CD8^+^ TILs treated with NPC-derived exosomes Supernatants of CD4^+^ TILs (A) and CD8^+^ TILs (B) from NPC tumor samples were harvested and used for a cytokine assay with the Bio-Plex cytokine assay system. TILs were firstly stimulated with OKT3 (2 μg/mL) and then treated or not with EXO1 or EXO2 in the presence or absence of the NPC cell line TW03; the corresponding supernatants were collected for cytokine assays. The cytokine concentrations (pg/mL) are reported as the mean ± S.E.M. of three experiments.

We hypothesized that EXOs from NPC cells would show a similar anti-inflammatory effect, consistent with their immunomodulation of T-cell proliferation and differentiation *in vitro*. Two CD8^+^ TIL and CD4^+^ TIL cell lines isolated from NPC specimens were cultured while stimulated or not with EXO1 or EXO2 in the presence or absence of NPC cells for 48 hours *in vitro*; the supernatant was harvested for cytokine profile detection. For CD4^+^ TILs, the proinflammatory cytokines IL1β, IL-6, and IL-10 but not IL-4 were increased when stimulated with EXO1 or EXO2; however, only the increase in IL-6 (without NPC cells) reached statistical significance (*P* < 0.05). Conversely, the other proinflammatory cytokines, TNFα, IL-12, GM-CSF, INFγ, IL-2, and IL-17, were decreased when stimulated with EXO1 or EXO2; however, only the decreases in IL-12, IL-17, and IL-2 (without NPC cells) and IFNγ (in the presence of EBV^+^ TW03 cells) reached statistical significance (P < 0.05). For CD8^+^ TILs, the change in proinflammatory cytokines was similar to that of CD4^+^ TILs when stimulated with EXO1 or EXO2. However, only the increases in IL-1β (in the presence of EBV^+^ TW03 cells) and IL-6 and IL-10 (in the presence of EBV^+^ TW03 cells) and the decrease in TNFα (without EBV^+^ TW03 cells) reached statistical significance (P < 0.05), as shown in Fig. 4.

### The enrichment of exosomal miRNAs down-regulated the MARK pathway in NPC

**Figure 5:**
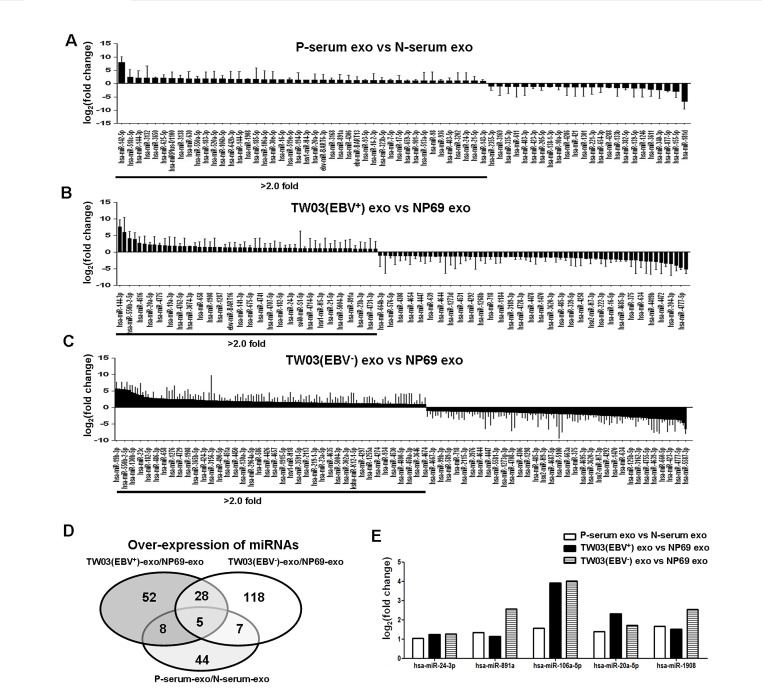
Differential miRNA expression in exosomes A. The mean fold changes of miRNAs in EXOs from the sera of NPC patients versus healthy controls (n = 10). B. The mean fold changes of miRNAs in EXOs from the NPC cell line TW03 (EBV^+^) versus the normal NP cell line NP69 (n = 3). C. The mean fold changes of miRNAs in EXOs from the NPC cell line TW03 (EBV^−^) versus the normal NP cell line NP69 (n=3). D. The number of over-expressed miRNAs in p-serum-EXOs/N-serum-EXOs, TW03 (EBV^+^)-EXOs/NP69-EXOs, or TW03 (EBV^−^)-EXOs/NP69-EXOs. E. The identification of five over-expressed miRNAs in P-serum-EXOs/N-serum-EXOs, TW03 (EBV^+^)-EXOs/NP69-EXOs, and TW03 (EBV^−^)-EXOs/NP69-EXOs: hsa-miR-24-3p, hsa-miR-891a, hsa-miR-106a-5p, hsa-miR-20a-5p, and hsa-miR-1908.

Exosomes released from tumor cells contain miRNAs in addition to exosomal proteins [36, 37]. Thus, we determined the presence of miRNAs in NPC tumor-derived exosomes by miRNA chip microarray analysis. We discovered that 326 and 321 miRNAs were enriched in the exosomes from the pooled sera of NPC patients (n = 10) and healthy controls (n = 10), respectively. In addition, 1511, 1686, and 1229 miRNAs were identified in the exosomes released from the NPC cell lines TW03 (EBV^+^) and TW03 (EBV^−^) and the normal NP cell line NP69, respectively. Moreover, of these, 68, 114, and 217 miRNAs were differentially expressed by more than 2-fold in NPC-derived exosomes from patient sera (P-serum) versus healthy control sera (N-serum) and from TW03 (EBV^+^) or TW03 (EBV^−^) versus NP69 cells, respectively, as shown in Figure 5A-C. A total of 44, 118, and 52 miRNAs were found to be over-expressed in the exosomes from P-serum, TW03 (EBV^+^), and TW03 (EBV^−^) compared with the exosomes from N-serum and NP69 cells, respectively (fold change > 2; Fig. 5D). Five miRNAs, including hsa-miR-24-3p, hsa-miR-891a, hsa-miR-106a-5p, hsa-miR-20a-5p, and hsa-miR-1908, were commonly over-expressed in the exosomes from P-serum and TW03 (EBV^+^) or TW03 (EBV^−^) cells (Fig. 5E).

**Fig 6:**
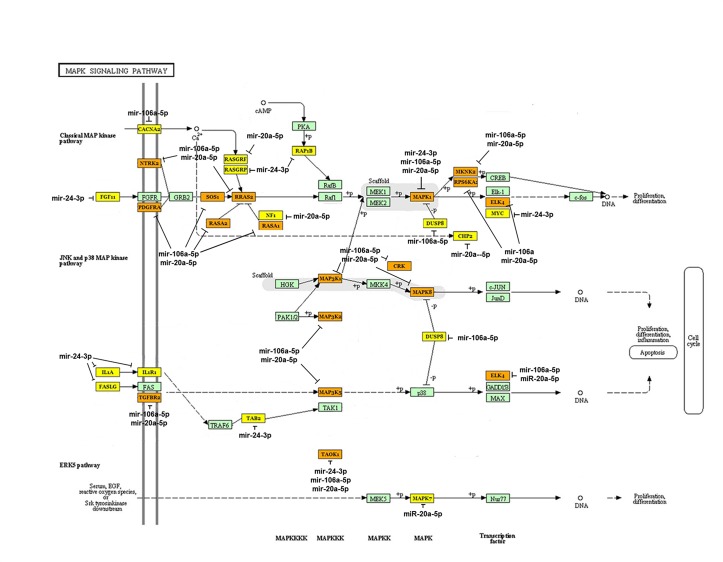
Bioinformatics analysis of the targets of the over-expressed miRNAs in NPC-derived exosomes The set of target genes that could be regulated by the five miRNAs predominantly expressed in NPC-derived exosomes were analyzed using the miRror program. Thirty-five genes were predicted by a combinatorial analysis using MicroT-CDS software. Network analysis of these genes using DIANA-mirPath v2.0 indicated the central involvement of the MAPK1 signaling pathway. The yellow-labeled genes are the targets of one miRNA of the five over-expressed miRNAs; the organ-labeled genes are the targets two of the miRNAs; the green-labeled targets are proteins in the MAPK1 pathway.

We analyzed the target genes and the signaling pathways regulated by the five over-expressed exosomal miRNA clusters in NPC tumor-derived exosomes using Kyoto Encyclopedia of Genes and Genomes (KEGG) and DIANA mirPath 2.0 software. These analyses resulted in a rank-ordered list of KEGG pathways, with statistical significance based on negative natural logged P-values (Table 1). Analyses of specific KEGG pathways targeted by the miRNAs were undertaken. The mitogen-activated protein kinase (MAPK) signaling pathway was associated with the smallest P-value (1.8×10^−11^) among the pathways targeted by the five miRNAs over-expressed in NPC exosomes, which included hsa-miR-24-3p, hsa-miR-891a, hsa-miR-106a-5p, hsa-miR-20a-5p, and hsa-miR-1908. In this pathway, more than one miRNA was often predicted to target MAPK1, TAOK3, NTRK2, PDGFRA, DUSP2, RASA2, ELK4, CRK, MAP3K1, RRAS2, TAOK1, RASA1, MAPK8, SOS1, MAP3K2, MKNK2, TGFBR2, MAP3K5, and PPP3R1, with the same gene potentially being targeted by several co-transcribed miRNAs (Fig. 6). For example, miR-20a-5p, miR-24-3p, and miR-106a-5p converge on MAPK1 and miR-20-5p and miR-106a-50 converge on TAOK3, demonstrating a combinatorial effect of miRNAs on the same target.

**Table 1:** Predicted KEGG pathways targeted by five over-expressed miRNAs

Up-regulated miRNAs	KEGG pathway	P-value	Number of genes
hsa-miR-24-3p, hsa-miR-891a, hsa-miR-106a-5p, hsa-miR-20a-5p, hsa-miR-1908-5p	MAPK signaling pathway	1.8×10^−11^	35
	Endocytosis	3.1×10^−8^	25
	Axon guidance	3.2×10^−8^	18
	Neurotrophin signaling pathway	6.6×10^−7^	15
	Circadian rhythm	1.2×10^−6^	8
	Hepatitis B	2.7×10^−5^	15
	Glycosphingolipid biosynthesis-ganglio series	3.0×10^−5^	4
	PI3K-Akt signaling pathway	7.1×10^−5^	28
	Colorectal cancer	1.6×10^−4^	11
	TGF-beta signaling pathway	1.8×10^−4^	11
	Pathways in cancer	2.8×10^−4^	34
	Osteoclast differentiation	6.8×10^−4^	10
	Ubiquitin mediated proteolysis	8.3×10^−4^	15
	Prostate cancer	8.5×10^−4^	11

## DISCUSSION

This study provides *in vivo* and *in vitro* evidence for exosome release from NPC cells, the clinical significance of the serum exosome concentration in NPC patients, the regulatory role of NPC-derived exosomes for T-cell dysfunction *in vitro*, the presence of miRNAs in exosomes from NPC patient sera and the NPC cell line TW03, and their regulated targets and signaling pathways. These findings have several clinical and immunopathological implications.

First, our data are the first to indicate that the serum exosome concentration is positively correlated with tumor lymph node metastasis and shorter disease-free survival in NPC patients. These findings not only indicate the clinical significance of exosomes in NPC patients but also suggest that exosomes play an important role in tumor progression in NPC. Given that the protein composi­tion of extracellular vesicles is similar to the parental cell type; tumor-derived extracellular vesicles can contain tumor-specific antigens and some oncoproteins and immunosuppressive molecules from the parallel tumor cells. For example, tumor-derived extracellular vesicles are enriched in CD95L, TRAIL, or galectin 9, which can promote T cell apoptosis [38-44]. Therefore, tumor-derived extracellular vesicles have been reported both to stimu­late and suppress tumor-specific and nonspecific immune responses. It has been reported that circulating exosome concentrations correlate with poorer survival and advanced disease stage in melanoma patients by educating bone marrow cells to support tumor growth and metastasis [45, 46]. The level of circulating exosomes has also been reported to correlate with poor prognosis parameters and shorter survival in colorectal cancer patients [47]. Some exosomal proteins or miRNAs have shown prognostic value in cancer patients, for example, exosomal ΔNp73 and exosomal miRNA in colon cancer, breast cancer, and lung cancer [36, 48-50]. Based on previous studies of exosomes in NPC and our data indicating the clinical significance and characterization of NPC exosomes [51], we assumed that tumor-derived exosomes could directly induce T-cell tumor antigen-specific and non-specific immune dysfunction, resulting in tumor lymph node transfer and immune escape in NPC.

Exosomes released from EBV-infected NPC cells can induce Th1 apoptosis through galectin-9/Tim-3 interaction [51]. However, that the modulations of T-cell immune responses, including proliferation, differentiation, and cytokine secretion in NPC are still not well described. In this study, we analyzed in detail the immunoregulation of T cells by NPC exosomes in several *in vitro* studies. Our observations showed that treatment with EXO1 or EXO2 inhibited the proliferation of OKT3-stimulated PBMCs, including CD4^+^ and CD8^+^ T-cells, and decreased ERK phosphorylation in EXO-stimulated T-cells compared with EXO-unstimulated T-cells. Moreover, treatment with EXO1 or EXO2 impeded the differentiation of Th1 and Th17 cells but promoted the differentiation of Tregs from naïve CD4+ T cells, as mediated by NPC cells. This modulatory impact of NPC-derived exosomes on T-cells may be associated with the alteration of the phosphorylation levels of the STAT1, STAT3, and STAT5 proteins. In addition, our multiplex cytokine array indicated that treatment with EXO1 or EXO2 could increase or decrease the secretion of different proinflammatory cytokines in CD4^+^ or CD8^+^ TILs from NPC specimens in the presence or absence of NPC cells *in vitro*. Overall, our results support the induction of T-cell dysfunction by tumor-derived exosomes, including antigen-specific and non-specific immune responses, by altering the proliferation, differentiation, and cytokine release of T cells in NPC. The presence of EBV antigens such as LMP1 on NPC exosomes (EXO1) was determined in our study and by others. However, our data showed that instead of augmenting immune responses, exosomes may ultimately also suppress antitumor immune responses in NPC. These observations may be associated with the presence of other immunosuppression-related molecules, such as galectin-9, TGFβ, and CXCR4, on NPC exosomes (Supplementary file Fig. S1). In line with our findings, increasing evidence shows that tumor-derived exosomes can induce tumor immune escape by impairing the function of immune cells, including T-cells, NK cells, and dendritic cells, through the enrichment of some biological proteins such as TGFβ or galectin-9 on exosomes in different cancers [16, 41, 42, 51-53].

Exosome-contained non-coding miRNAs can be transferred into tumor cells or immune cells to affect cellular gene expression and cell behavior, including the strengthening of the stemness of tumor cells [54-56]. In the present study, we identified the presence of miRNAs in exosomes from the sera of NPC patients and healthy donors and from TW03 cells (EBV^+^), TW03 cells (EBV^−^), and NP69 cells using a chip microarray analysis. Our results showed that five exosomal miRNA clusters, including hsa-miR-24-3p, hsa-miR-891a, hsa-miR-106a-5p, hsa-miR-20a-5p, and hsa-miR-1908, were abundant in NPC tumor-derived exosomes from patient sera or TW03 cell lines versus the exosomes from healthy donor sera or NP69 cells. According to analyses using KEGG and DIANA mirPath 2.0 software, these miRNAs can down-regulate many signaling pathways (Table 1). Of these, the mitogen-activated protein kinase (MAPK) signaling pathway, which regulates the proliferation and differentiation of cells, had the smallest natural logarithm P-value (1.8×10^−11^) and was down-regulated by the 5 over-expressed miRNA clusters in NPC exosomes. In addition, 35 genes in the MAPK pathway are targeted by one or more miRNAs. This finding supports that the alteration of T-cell proliferation and differentiation by NPC-derived exosomes is associated with these five enriched miRNAs, perhaps via the phosphorylation of ERK and STAT proteins through the MAPK pathway. Another study noted that hsa-miR-20a-5p targets the JAK1 gene to regulate the JAK/STAT signaling pathway [57]. This is consistent with our findings indicating that exosomes alter the phosphorylation of STAT proteins in tumor-induced CD4^+^ T cells to affect T-cell differentiation and that hsa-miR-20a-5p was over-expressed in NPC exosomes. Future studies are planned to examine the effect of the enrichment of exosomal miRNAs on target genes and T-cell functions.

In conclusion, the findings of the present study provide novel insight into the role of tumor-derived exosomes in disease progression and the regulation of T-cell function *in vivo* and *in vitro*. Our data indicate that the serum exosome concentration has clinical relevance and prognostic value in NPC patients. NPC tumor-derived exosomes mediate T-cell dysfunction, including proliferation, differentiation, and cytokine secretion, which might be associated with the enrichment of exosomal miRNAs targeting the down-regulation of the MAPK1 and JAK/STAT pathways. These findings provide novel clarification of tumor immune evasion and potential targets for NPC immunotherapy.

## MATERIALS AND METHODS

### Patients and cell lines

Serum was collected from 83 newly diagnosed NPC patients at Sun Yat-sen University Cancer Center (supplemental Table 1) from 2011 to 2013. Serum and peripheral blood mononuclear cells (PBMCs) from blood samples obtained from healthy individuals were isolated and then frozen for *in vitro* proliferation and differentiation analyses. P109CD4^+^ TIL and P125CD8^+^ TIL cell lines were isolated from NPC tissues and maintained in low-dose IL-2 (300 IU/mL) medium. NPC tumor cell lines, including TW03 (EBV^+^) and TW03 (EBV^−^), were maintained in RPMI 1640 medium containing 10% fetal bovine serum (FBS). The normal nasopharyngeal (NP) cell line NP69 was maintained in keratinocyte-SFM medium (Invitrogen). This study was conducted in accordance with the Helsinki Declaration; all patients and healthy controls signed a consent form approved by the Research Ethics Committee of the Sun Yat-sen University Cancer Center.

### Reagents and antibodies

Aldehyde/sulfate latex beads (C37253) and carboxyfluorescein diacetate succinimidyl ester (CSFE) were purchased from Life Technologies. The BCA Protein Assay kit (23227) and the ECL detection kit (34075) were obtained from Thermo Scientific (Hudson, NH, USA). A multiplex ELISA kit Bio-Plex Pro™ was purchased from Bio-Rad Laboratories (Hercules, CA, USA). For flow cytometric analyses, antibodies (Abs) against the following human proteins obtained from eBio­science were used: CD8 (12-0088-42), CD39 (11-0399-42), CD73 (11-0739-42), CXCR4 (25-9999-42), IFN-γ (17-7319-82), and Foxp3 (53-4777-73). Antibodies against the following human proteins obtained from BD Biosciences were also used: CD4 (555346), IL-17a (560436), HLA-DR (555812), and LAMP-1 (555801). An antibody against human galectin-9 (348903) was purchased from Biolegend. An antibody against human TGF-β (348903) was purchased from R&D Systems. Mouse IgG1 antibody (sc-65218) was purchased from Santa Cruz.

For Western blot analyses, anti-P-ERK1/2 (9101), anti-P-p65 (3033), and anti-P-STAT3 (9131) were purchased from Cell Signaling Technology. Anti-P-STAT1 (3324) and anti-P-STAT5 (1208) were obtained from Epitomics. Anti-LMP1 (M0897) was obtained from DAKO, and anti-human MHCI (NB500-304) was obtained from Novus Biologicals. Anti-CD63 (sc-5275) was purchased from Santa Cruz Biotechnology, and anti-human CytC (PR-0235) was purchased from ZSGB-BIO.

### Preparation and quantification of exosomes

TW03 (EBV^+^) and TW03 (EBV^−^) were cultured in 1% exosome-free RPMI 1640 complete medium for 48 h for exosome isolation. Exosomes in NPC cell culture supernatants or from the plasma of NPC patients were isolated as previously described [58]. Briefly, collected culture supernatants were differentially centrifuged at 300 × g for 10 min; 1,200 × g for 20 min; and 10,000 × g for 30 min at 4°C. Subsequently, the supernatant was filtered (0.22 μM Millex GP) and ultracentrifuged at 100,000 × g for 1 h at 4°C. After removing the supernatant, the exosome pellets were washed in a large volume of ice-cold PBS and centrifuged at 100,000 × g for another 1 h at 4°C, resuspended with PBS, and stored at -80°C until use. Exosomes were quantified by measuring total protein (Pierce BCA Protein Assay).

### Characterization of exosomes by electron microscopy, FACS, and Western blot analysis

For electron microscopy visualization of exosomes, 3 μl of exosomes suspended in PBS was placed on a glow-discharged Formvar carbon-coated grid and negatively stained with 2% uranyl acetate solution. Images were obtained using an FEI Tecnai F20 electron microscope operated at 200 kV, and images were captured using a 4k x 4k CCD camera. The size of the particles was assessed by NanoSight particle tracking (NanoSight Ltd.). Particles of 30-120 nm were designated as exosomes. For FACS analysis, the exosomes were coated onto aldehyde/sulfate latex beads (1% solids, Invitrogen) at 4°C overnight; the reaction was stopped with 100 mM glycine. The exosome-coated beads were washed thrice and resuspended in PBS. The beads were then incubated with the corresponding fluorescent Abs for 1 h at room temperature in the dark. The beads were analyzed by flow cytometry using an FC500 flow cytometer, and the obtained data were analyzed with CXP software (Beckman Coulter).

For Western blot analyses, cell lysates or exosomes (20 μg of protein) were separated by 8% or 10% SDS-PAGE, transferred onto PVDF membranes (Millipore), blocked, and incubated with the different primary Abs described above followed by HRP-conjugated secondary Abs (Santa Cruz). The protein bands were visualized using an ECL detection kit (PerkinElmer Life Science).

### T-cell proliferation and differentiation assay

PBMCs from healthy donors were labeled with carboxyfluorescein diacetate succinimidyl ester (CSFE, 10 μM). The CSFE-labeled PBMCs were plated in OKT3-coated 96-well plates and treated with 10 μg/mL exosomes or left untreated, followed by culturing for 5 days. PBMCs were harvested and stained for CD3, CD4, and CD8 cell surface markers; the data were acquired and detected by FACS.

Naïve CD4^+^ T cells were cultured in T cell medium containing 100 IU/mL IL-2 and 10% FBS at a concentration of 1×10^5^ cells/well in a 48-well plate and stimulated with plate-bound OKT3 (1 μg/mL). The cells were co-cultured with irradiated tumor cell lines at a 1:1 ratio with or without 10 μg/mL exosomes for 7 days. Half of the medium was replaced with fresh medium on days 3 and 6. After 7 days, the percentages of Th1 and Th17 cells and Tregs were determined by FACS analysis.

### Cytokine assay using NPC-derived exosome-treated T cells

A multiplex ELISA kit (Bio-Plex Pro Assays, Bio-Rad) was used to measure the level of 10 cytokines in culture supernatants. Two NPC TIL cell lines, P109 TILs (CD4^+^) and P125 TILs (CD8^+^), were generated and maintained in our lab. CD4^+^ TILs or CD8^+^ TILs were plated into an OKT3-coated 24-well culture plate at 1×10^6^/mL and left untreated or stimulated with 10 μg of NPC-derived exosomes in the presence or absence of the NPC cell line TW03 overnight. The supernatants were harvested for cytokine detection.

### Exosomal microRNA chip array

Identification of microRNAs was performed with the Exiqon Array platform. In brief, total RNA was isolated using TRIzol (Invitrogen) and the miRNeasy mini kit (QIAGEN) according to the manufacturers' instructions. The RNA was quantified and assessed using a NanoDrop spectrophotometer (ND-1000, NanoDrop Technologies). After the isolation of RNA from the samples, the miRCURY™ Hy3™/Hy5™ Power labeling kit (Exiqon) was used according to the manufacturer's guidelines for miRNA labeling. After labeling, the Hy3^™-^ labeled samples were loaded onto the miRCURY™ LNA Array (v.16.0 or v18.0, Exiqon) according to the array manual. In brief, the total 25 μL mixture from the Hy3^™-^ labeled samples in 25 μL of hybridization buffer was first denatured and then incubated on ice for 2 min; the mixture was hybridized to the microarray for 16-20 h at 56°C in a 12-Bay Hybridization System (Hybridization System - Nimblegen Systems). Following hybridization, the slides were washed thrice and dried by centrifugation. The microarray slides were then scanned using the Axon GenePix 4000B microarray scanner, and the scanned images were imported into GenePix Pro 6.0 software (Axon) for grid alignment and data extraction. Replicated miRNAs were averaged, and miRNAs with intensities ≥ 30 in all of the samples were chosen for calculating the normalization factor. The expression data were normalized using median normalization. After normalization, the differentially expressed miRNAs were identified using fold-change filtering. The threshold value for significance used to define the up-regulation or down-regulation of miRNAs was a fold change > 2.0. The microarray data were deposited in the NCBI Gene Expression Omnibus (GEO) under accession codes GSE57319 and GSE57367.

### Bioinformatics analysis of miRNA target genes and pathways

To examine the biological function of the five over-expressed miRNAs, we used the DIANA mirPath2.0 software, which combines a common target prediction algorithm (DIANA-microT-CDS) and the Kyoto Encyclopedia of Genes and Genomes (KEGG) pathways to analyze the combinatorial effect of different miRNAs. The target prediction threshold of DIANA-microT-CDS was set at 0.85. The results consisted of selective KEGG pathways, P-values, and the number of genes. The P-value threshold was set at 0.0001.

### Statistical analysis

All analyses were performed with SPSS 13.0. Numerical data are presented as the mean ± standard error of the mean (SEM). A standard two-tailed Student's t-test and a paired Student's t-test were used for comparison of the numerical data, and P-values less than 0.05 were considered significant.

## SUPPLEMENTARY MATERIAL FIGURE AND TABLE


