# Supplementary material for: Tumor-derived exosomes promote tumor progression and T-cell dysfunction through the regulation of enriched exosomal microRNAs in human nasopharyngeal carcinoma
Source: Oncotarget. 2014 Jun 19;5(14):5439–52. doi: 10.18632/oncotarget.2118 (PMC4170615; doi:10.18632/oncotarget.2118)
Supplement: Supplementary file 1 [file oncotarget-05-5439-s001.pdf]

Tumor-derived exosomes promote tumor progression and T-cell dysfunction through the regulation of enriched exosomal microRNAs in human nasopharyngeal carcinoma

Supplementary Material

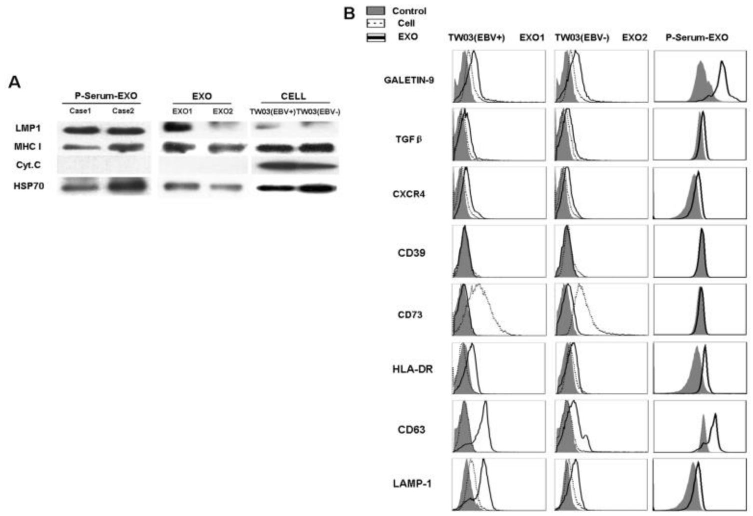

Figure S1: Phenotypic analysis of NPC-derived exosomes. A. Representative western blot of LMP1, HLA class I, Cyt. C and HSP70 (control) in NPC tumor cells and NPC-derived exosomes. B. NPC TW03 (EBV^{+}) and TW03 (EBV^{-}) cells and TW03-derived exosomes (EXO1, EXO2) and serum exosomes from NPC patient were stained with a panel of antibodies (Abs) and then analyzed by flow cytometry. The cells and EXOs were also stained with isotype control Abs and employed as control populations. One representative experiment of three is displayed.

Table S1 Clinical characteristics of 83 patients with nasopharyngeal carcinoma

Characteristics	No. (%)
**Total cases**	83
**Age, years**	
Median	46
Range	22-75
**Gender**	
Male	70 (87.0%)
Female	13 (13.0%)
**Tumor (T) status**	
T1	2 (2.4%)
T2	19 (22.9%)
T3	44 (53.0%)
T4a-b	18 (21.7%)
**Lymph node (N) status**	
N0	13 (15.7%)
N1a-b	41 (49.4%)
N2	22 (26.5%)
N3	7 (8.4%)
**Distant metastasis (M) status**	
M0	82 (98.8%)
M1	1 (1.2%)
**TNM stage**	
I	0 (0.0%)
II	11 (13.3%)
III	51 (61.4%)
IVa-b	21 (25.3%)
**Recurrence**	
No	77 (92.8%)
Yes	6 (7.2%)
**Death**	
No	81 (97.7%)
Yes	2 (2.3%)
